# Double versus single T-tube drainage for frank cysto-biliary communication in patients with hepatic cystic echinococcosis: a retrospective cohort study with median 11 years follow-up

**DOI:** 10.1186/s12893-020-01028-8

**Published:** 2021-01-06

**Authors:** Paizula Shalayiadang, Tiemin Jiang, Yusufu Yimiti, Bo Ran, Abudusalamu Aini, Ruiqing Zhang, Qiang Guo, Ayifuhan Ahan, Abuduaini Abulizi, Hao Wen, Yingmei Shao, Tuerganaili Aji

**Affiliations:** 1 Hepatobiliary and Echinococcosis Surgery Department, Digestive and Vascular Surgery Center, First Affiliated Hospital of Xinjiang Medical University, #137 South Liyushan Road, Urumqi, 830054 China; 2 Xinjiang Uyghur Autonomous Region Clinical Research Center for Echinococcosis and Hepatobiliary Diseases, First Affiliated Hospital of Xinjiang Medical University, Urumqi, China; 3 Digestive and Vascular Surgery Center, First Affiliated Hospital of Xinjiang Medical University, Urumqi, China; 4 State Key Laboratory of Pathogenesis, Prevention and Management of High Incidence Diseases in Central Asia, Xinjiang Medical University, Urumqi, China; 5 WHO Collaboration Center on Prevention and Management of Echinococcosis, Clinical Medical Institute, First Affiliated Hospital of Xinjiang Medical University, Urumqi, China; 6 Xinjiang Uyghur Autonomous Region Key Laboratory of Echinococcosis, Clinical Medical Institute, First Affiliated Hospital of Xinjiang Medical University, Urumqi, China

**Keywords:** Cystic echinococcosis, Frank cysto-biliary communication (FCBC), Intra-biliary cyst rupture, Biliary fistula, T-tube drainage, Biliary complication, Follow-up

## Abstract

**Background:**

Partial peri-cystectomy (PPC) is one of the major surgical approaches for hepatic cystic echinococcosis (CE) and has been practiced in most centers worldwide. Cysto-biliary communication (fistula, leakage, rupture) is a problematic issue in CE patients. T-tube is a useful technique in situations where an exploration and decompression are needed for common bile duct (CBD). However, postoperative biliary complications for cystic cavity still remains to be studied in depth.

**Methods:**

A retrospective cohort analysis of CE cases in our single center database from 2007 March to 2012 December was performed. Patients (n = 51) were divided into two cohorts: double T-tube drainage (one at CBD for decompression and one at the fistula for sustaining in cystic cavity, n = 23) group and single T-tube drainage cohort (only one at CBD for decompression, n = 28). Short-/long-term postoperative complications focusing on biliary system was recorded in detail and they were followed-up for median 11 years.

**Results:**

Overall biliary complication rates for double and single T-tube drainages were 17.4% vs. 39.3% (*P* > 0.05). Short-term complications ranged from minor to major leakages, cavity infection and abscess formation, and prevalence was 17.4% vs. 21.4% (*P* > 0.05) respectively for double and single T-tube groups; most importantly, double T-tube drainage group had obvious advantages regarding long-term complications (*P* < 0.05), which was biliary stricture needing surgery and it was observed only in single T-tube drainage group.

**Conclusions:**

Double T-tube drainage had better outcomes without procedure-specific postoperative biliary complications than single T-tube drainage. Meanwhile, we recommend long-term follow-up when comparing residual cavity related biliary complications in CE patients as it could happen lately.

## Background

Human cystic echinococcosis (CE) in one of the lethal infectious diseases and causes severe organ damage [1, 2]. Mortality of echinococcosis is > 90% within 10–15 years if left untreated or inadequately treated after initial diagnosis. Radical resections, such as total peri-cystectomy with non-opened cyst and liver resection are considered as the best curable options [2, 3]. However, not all cases indicate radical approach due to crucial cyst location or corresponding complications, needing non-radical surgeries.

The inflating growth pattern of CE within the liver often leads to various vasculature comorbidities [1, 4, 5]. Some of them presents certain therapeutic challenges for surgeons and consumes patience of the subjects by lowering life quality. Cysto-biliary complications (fistula, leakage, rupture) is one of the most frequent complications [4, 6]. According to reports, communication between cystic cavity and the intra-hepatic biliary tracts could be classified as major (> 5 mm in diameter), minor (< 5 mm in diameter) and invisible (occult, hard to observe with naked eye but definitely exists). Invisible or occult communication occurs in 10–37% of CE patients, however, frank cysto-biliary communication (FCBC) is an open intercommunication between the cystic cavity and intra-hepatic bile ducts that allows the contents of the cyst to drain directly into the bile duct as well as biliary poring into the cystic cavity [7]. FCBC could cause obstructive jaundice, cholangitis, cystic infection, gastrointestinal discomfort and naplulaxis [8].

Therefore, cholecystectomy, CBD exploration as well as T-tube drainage has been practiced after partial peri-cystectomy (PPC) to eradicate intra-biliary debris and decompression, and gradually became routine procedure [9, 10]. In this situation, the ruptured site was managed by suturing, hepatectomy of relevant liver parenchyma, residual cavity drainage, omentoplasty etc., however, postoperative complications still draw attention [9–12]. Long term follow-up after this surgical approach has revealed potentially serious postoperative complications, including biliary stricture, biliary fistula, wound infection as well as abscess formation [5, 6]. Additionally, most health care professionals have limited experience with such situations outside the endemic region.

More than a decade ago in our center, extra T-tube was introduced as a sustaining method of the FCBC site in order to achieve better outcome. Initially, it was an evolutionary process: non-T-tube catheter and endo-drainage tubes were placed in the cystic cavity for leakage drainage, and it evolved gradually into T-tube. Our first cohort was specially designed to compare biliary outcomes in such patients from 2007 March. Currently, the follow-up was finished with comparable prognosis, and we thought it would be helpful for academic society and infectious disease professionals.

## Methods

**Fig. 1:**
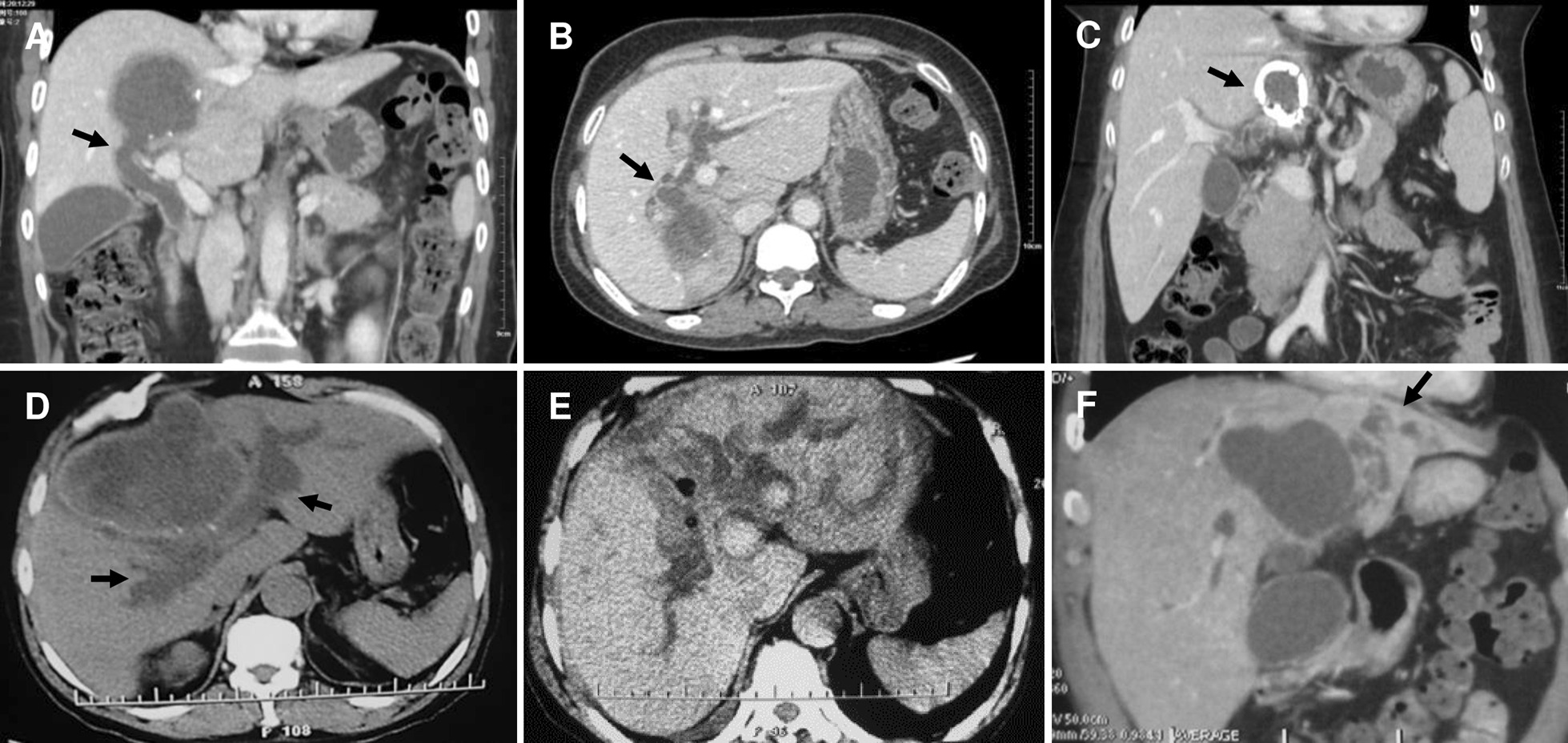
CT presentations of frank cysto-biliary communications. **a** Cysto-biliary communication (arrow) at right supra section and debris-filled common bile duct; **b** Cysto-biliary communication (arrow) at right posterior lobe; **c** Capsule calcified cyst (arrow) interlinking with left hepatic duct; **d** Medial lobular cyst presenting cysto-biliary communication to major hepatic ducts form both sides (arrows); **e** Severe left lateral lobe liver damage caused by cysto-biliary communication; **f** Hepato-atrophy (arrow) of left lateral lobe led by cysto-biliary communication

**Fig. 2:**
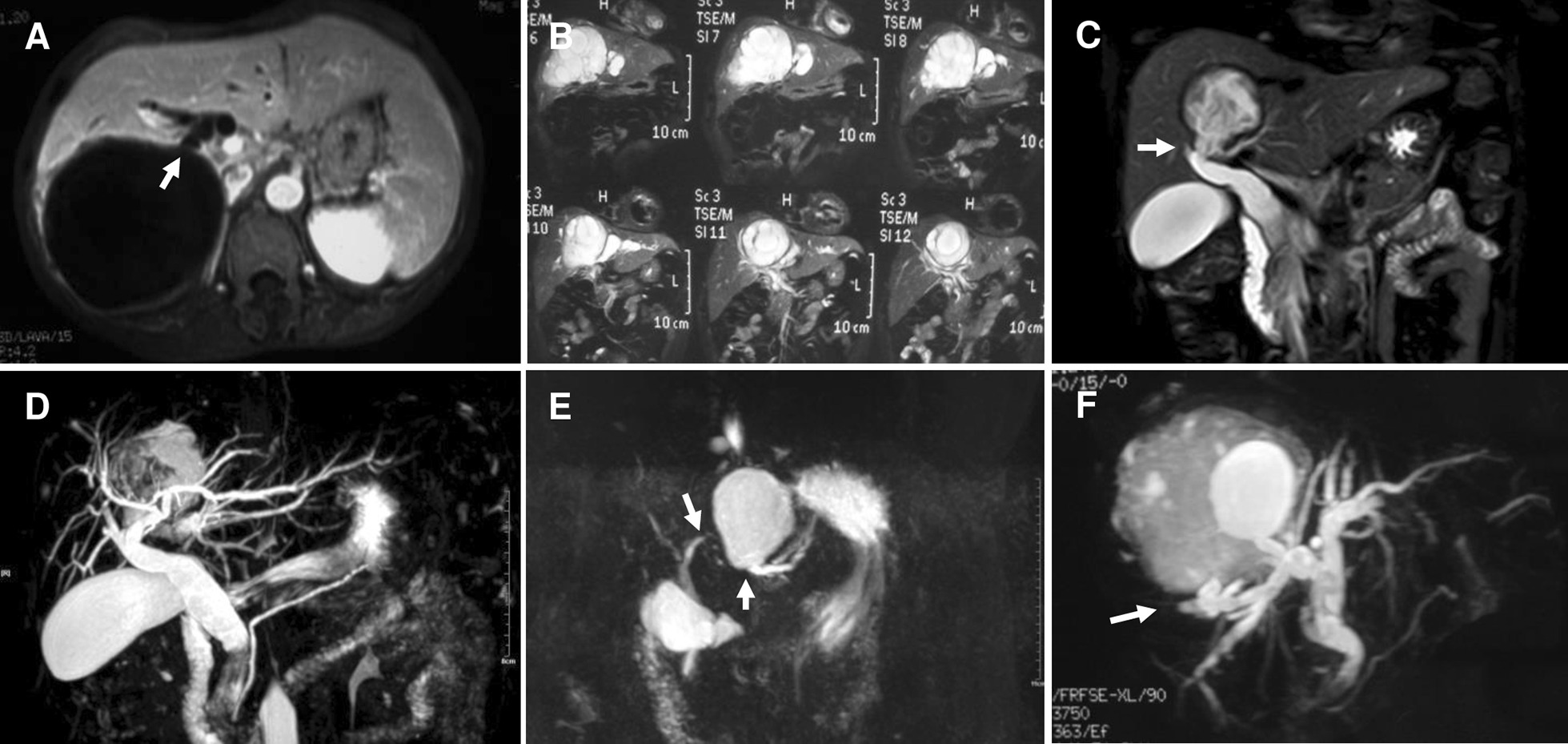
MR/MRCP manifestations of frank cysto-biliary communications. **a** Cysto-biliary communication (arrow) at right posterior lobe; **b** cysto-biliary communication of “nested” cyst at main ductal branches of the liver; **c** cysto-biliary communication (arrow) at right supra section; **d** cysto-biliary communication (arrow) at right posterior lobe; **e** left lateral lobular cyst that played as “drainage pool” of the bile via cysto-biliary communication (lower arrow), note that proximal end of left hepatic duct was cramped (upper arrow) due to functional disuse; **f** cysto-biliary communication (arrow) at right lobe and biliary disuse of superior tributary

**Fig. 3:**
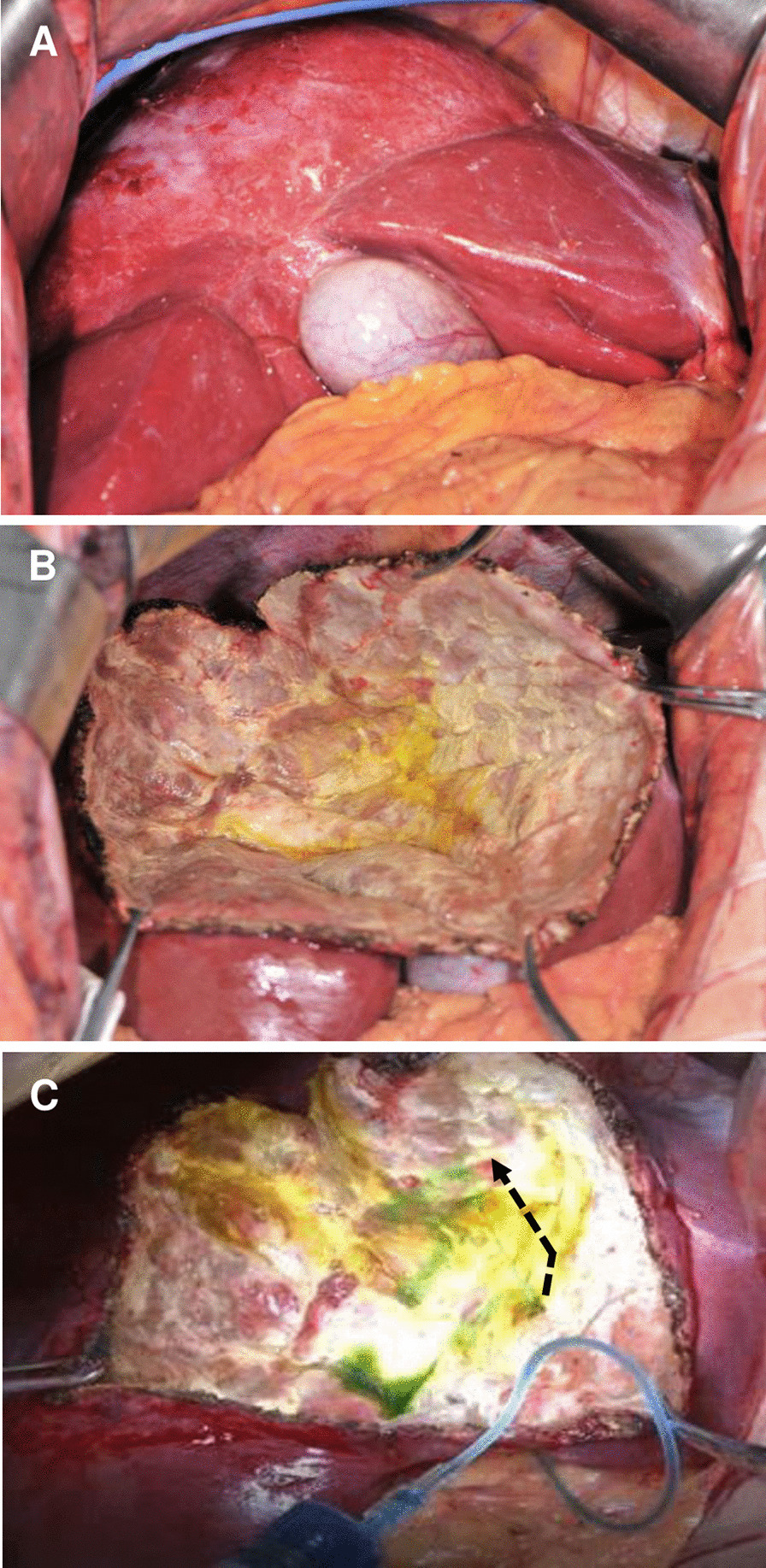
Example of intraoperative technique to discover cysto-biliary communication. **a** Surgical exposure of large echinococcal cyst; **b** posing cystic cavity for potential biliary leakage after partial peri-cystectomy plus total endo-cystectomy; **c** liquid spray (dotted arrow) form leakage site when injecting methylene blue (1:250 diluted with normal saline)

From 2007 March to 2012 December, altogether 660 hepatic CE patients have been hospitalized in our center, and 51 of which (3.5%) without hepatobiliary surgical history underwent PPC in our center due to CE with FCBC. Their most common complaints were abdominal pain, jaundice, nausea and fever. Patient demographics, cyst features and clinical symptoms were presented in Table 1. Preoperative computed tomography (CT) and magnetic resonance imaging and cholangiopancreatography (MR/MRCP) results indicated that, the certain bile duct where the rupture occurred, mostly were perihilar lobular bile ducts (Figs. 1 and 2). PPC was performed in all patients; debris in biliary tracts was removed through CBD using choledochoscopy after cholecystectomy; biliary tract was explored by injecting methylene blue (1:250 dilution with normal saline) to discover the FCBC site (Fig. 3). Owing to the basis of FCBC, setting decompression T-tube and sustaining T-tube drainage have been introduced. Consequently, we retrospectively divided these special subjects into two groups based on their operative procedures (double T-tube drainage group and single T-tube drainage group, specifically mentioned blow), forming this research cohort.

**Table 1:** Preoperative patient demographics, cyst features and clinical symptoms

Groups	Double T-tube drainage	Single T-tube drainage with sutured fistula	Total	*P* value
Sample size	23	28	51	–
Gender (male/female)	13/10	17/11	30/21	0.7621
Age (median with range)	46 (29–65)	49.5 (33–66)	47 (29–66)	0.1529
Cyst types (CE2/CE3/CE4)^a^	8/9/6	12/10/6	20/19/12	0.5642
Cyst location (RL/LL)	18/5	25/3	43/8	0.2814
FCBC site (1st/2nd intrahepatic biliary tree)	15/6	20/8	28/21	1.0000
Cyst size (median with range, cm)	10.3 (6.3–18.0)	8.1 (6.0–16.5)	9.1 (6.0–18.0)	0.0799
Cyst capsule (non-calcified/calcified)	17/6	23/5	40/11	0.4771
Direct bilirubin elevation (yes/no)	19/4	22/6	41/10	0.7178
Symptoms (symptom with frequency)	Abdominal pain/distention (21) Jaundice (18) Fever/Chill (9) Nausea/vomiting (8)	Abdominal pain/distention (25) Jaundice (19) Fever/Chill (12) Nausea/vomiting (7)	Abdominal pain/distention (46) Jaundice (37) Fever/Chill (21) Nausea/vomiting (15)	Abdominal pain/distention: 0.8094 Jaundice: 0.4074 Fever/Chill: 0.7879 Nausea/vomiting: 0.4455

^a^Classified as WHO-IWGE classifications

*RL* right lobe, *LL* left lobe

### Double T-tube drainage

**Fig. 4:**
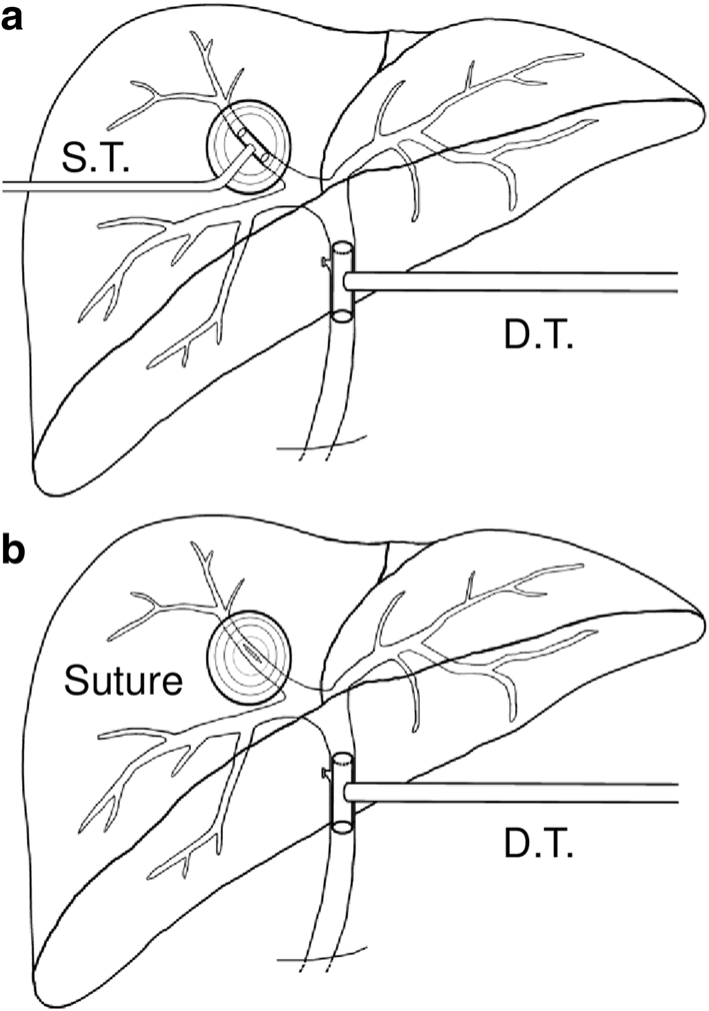
Schematic diagram of different biliary surgical techniques for cysto-biliary communications. **a** Double T-tube drainage due to fistula; **b** Single T-tube drainage with sutured fistula. (D.T. = decompression T-tube at common bile duct; S.T. = sustaining T-tube at cysto-biliary communication site; Suture = suturing of the cysto-biliary communication site; Note that there were routine abdominal/cystic cavity draining catheters within these two techniques but they were omitted in order to magnify T-tubes in the figure)

Schematic diagram (Fig. 4a) presented this procedure that was performed in 23 patients. In this method, main operation steps included: (i) PPC followed by exploration of inside surface of remnant peri-cyst to discover potential ruptured bile ducts; (ii) cholecystectomy, choledotomy and choledochoscopic exploration, removal of feces or debris in biliary tracts, biliary tree testing by infusing methylene blue through CBD, minor leakages in the residual cavity were sutured by using 4-0 or 5-0 absorbable sutures; (iii) upon exposuring FCBC site, one sustaining T-tube was placed where it ruptured, followed by a regular decompression T-tube at the CBD was introduced, thereafter, the rupture and the biliary tracs were reexamined by flushing methylene blue through decompression T-tube until no leakage was noticed; (iv) after careful examination and assuring patency of biliary tree, other two routine catheters were placed respectively at the residual cavity and the hepatic hilar region for post-surgical observation of any bile leakage or infections.

### Single T-tube drainage with sutured fistula

Schematic diagram (Fig. 4b) showed this procedure that was performed in 28 patients. Main steps (i), (ii) and (iv) were same with above approach; (iii) the specific ruptured bile duct was sutured using 5-0 or 6-0 absorbable sutures, then a regular decompression T-tube at CBD was introduced, thereafter, reexamination by flushing methylene blue through T-tube was performed until no leakage was noticed.

### Postoperative T-tube removal

**Fig. 5:**
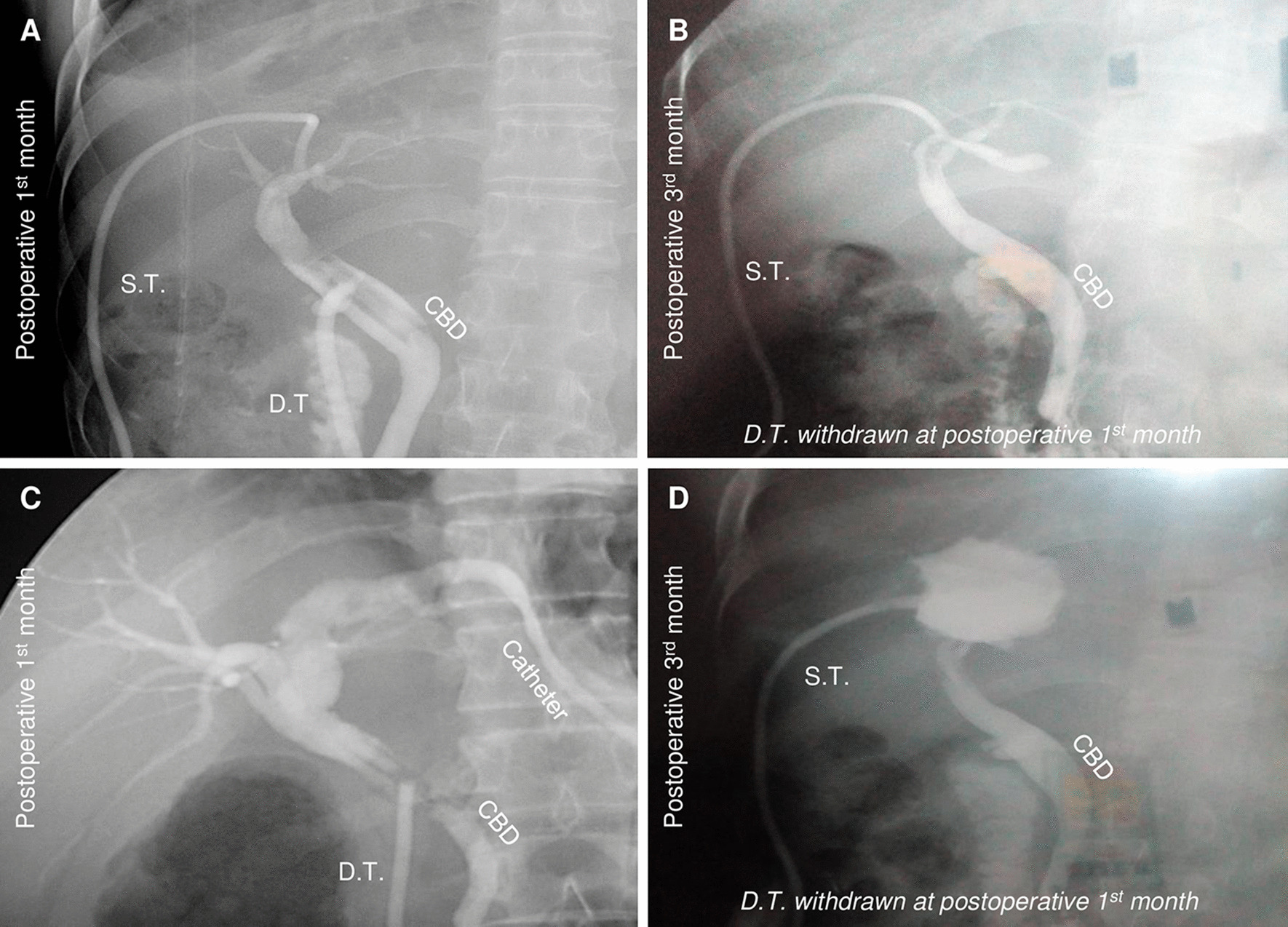
Postoperative cholangiograms for the tubes/catheters. **a** Satisfactory drainage without obvious leakage at postoperative first month (corresponding to *double T-tube drainage*); **b** Satisfactory healing of the cavity without apparent leakage at postoperative third month after withdrawal of decompression T-tube at postoperative one month (corresponding to *double T-tube drainage* for the same patient in **a** in this figure); **c** Qualified drainage without obvious leakage at postoperative first month (corresponding to *single T-tube drainage with sutured fistula*); **d** unsatisfactory outcome of the original leakage in cystic cavity at postoperative third month, note that this leakage had not been observed at postoperative first month, which indicated late leakage at original cysto-biliary communication site after withdrawal of decompression T-tube at first month (corresponding to *single T-tube drainage with sutured fistula*)

All subjects hold decompression T-tubes for about one month after cholangiographic examination was negative in both groups; and all sustaining T-tubes were removed if the results showed no leakage and indicates fully recover with the help of cholangiography at three months after the surgery in double T-tube drainage group (Fig. 5).

### Patient follow-up and data analysis

Subject were prescribed with albendazole at the dosage of daily 15 mg/kg and followed-up after discharge from hospital at every 3–6 months for the first 2 years and at every 1–2 year afterwards. Follow-up results were obtained by visiting or interviewing through phone contacts. Their clinical data was analyzed, including demographic characteristics, cystic lesion features, operative methods, hospital-stay, short-/long-term biliary complications. Mann–Whitney and Chi-Square tests were used to compare statistical data between groups.

## Results

In general, there were no mortality and no malignancy observed in any of these patients. In terms of prognosis, altogether 36 (70.6%) patients achieved clinical cure eventually, and other 15 (29.4%) cases suffered from short-/long-term complications, but equally reached clinical cure after corresponding surgical interventions. Short-/long-term complications rates were 19.6% (10/51) and 9.8% (5/51) respectively. Postoperative complications between the two groups were compared in Table 2. Single T-tube group stayed in hospital longer than double T-tube group before discharge (*P* < 0.05).

**Table 2:** Surgery and surgical outcome of double versus single T-tube drainages in particular with biliary complications

Items	Double T-tube drainage	Single T-tube drainage with sutured fistula	Total	*P* value
Common procedures	Partial peri-cystectomy + total endo-cystectomy + decompression T-tube drainage at CBD + cystic cavity draining catheter + abdominal cavity draining catheter		–	–
Differential techniques	Sustaining T-tube drainage at fistula	Fistula suture	–	–
Length of stay (days)	9 (7–13)	11 (9–17)	9 (7–17)	< 0.0001
Postoperative complication rate (short/long/overall, %)	17.4/0/17.4	21.4/17.9/39.3	19.6/9.8/29.4	0.7178/0.0329/0.0877
Short term complication (frequency)	Minor leakage (2) Cavity infection (2)	Minor leakage (3) Major leakage (1) Cavity infection (1) Abscess formation (1)	Minor leakage (5) Major leakage (1) Cavity infection (3) Abscess formation (1)	Minor leakage: 0.8094 Major leakage: 0.3600 Cavity infection: 0.4390 Abscess formation: 0.3600
Long term complication (frequency)	–	Biliary stricture (5)	Biliary stricture (5)	0.0329
Follow-up Duration (years)	10.6 (7.3–13.3)	11.3 (8.4–13.8)	11.0 (7.3–13.8)	0.1583

*CBD* common bile duct

### Short term outcomes

(a) Minor biliary fistula (≤ 250 ml/24 h) was observed in 3 (10.7%) cases in single T-tube group, while in 2 (8.7%) in double T-tube group, and the former three ceased within 1 week by extended peritoneal drainage, but the latter two stopped with longer duration of T-tube except from extended peritoneal drainage. (b) One case (3.6%) in single T-tube group struggled from major biliary fistula (> 250 ml/24 h), unfortunately, the case developed into peritonitis and received reoperation with peritoneal lavage and debridement. (c) Infection of cavity was occurred in 1 (3.6%) patient in single T-tube group, which was managed by wound care and debridement. Whereas, in double T-tube group, 2 (8.7%) subjects with infection of incisions were treated by antibiotic therapy and wound care by meticulous dressing and debridement. (d) Abscess formation was only discovered in 1 (3.57%) case in single T-tube group, which was treated through percutaneous drainage.

### Long term outcomes

In the long term, no complications were noticed in double T-tube group, however, biliary stricture was discovered in five cases in single T-tube group during 2–9 years postoperatively (*P* < 0.05). Out of these patients, two of them accomplished dilation of biliary stenosis and cured thanks to decompression from percutaneous cholangiography and drainage (PTCD) of upper biliary trees. While, two of them recovered through reoperation with continuous T-tube sustaining which took half year to heal. Another one case had to receive left hepatectomy to stop continues biliary stenosis after 2 years.

## Discussion

FCBC with overt passage of intra-cystic material to the biliary tract is a serious complication, and the reported frequency is from 3 to 37% in different case series [6, 12]. Intrabiliary rupture is mainly resulted from gradually increasing cysts, causing bile stasis. The increased intraductal pressure induces fissure formation in the duct wall while local intracystic water pressure (up to 80 cm) lead to the rupture of the cyst into biliary tract [13]. FCBC is majorly established in centrally localized cysts near to the hilum, especially in liver segments III, IV, V, VI, and the possibility will increase for larger cysts (> 10 cm) [9, 14, 15]. The main surgical rule for hydatid cyst with intrabiliary rupture should be: cystic content evacuation, cavity management, clearance of cystic material from biliary tract to assure normal biliary flow. Although, there are different opinions on cavity management, but one core rule is: CBD exploration with clearance of cystic material and maintenance of normal biliary channel.

For attaining proper cavity management with proper healing of FCBC, biliary tract decompression is fundamental. Various techniques including choledochoduodenostomy, T-tube drainage, and transduodenal sphincteroplasty have been recommended to decompress intrabiliary pressure [5, 11]. Placement of T-tube enables easier monitoring and seems to be traditional method after intrabiliary rupture of CE. But the usage of T-tube is not free of complications. The most frequently encountered morbidities were external biliary fistulas and suppuration of the residual cavity and postoperative biliary stricture. To prevent these late complications (such as stricture) after PPC and T-tube decompression, we further performed extra T-tube in the cysto-biliary orifice of bile duct in residual cavity. However, since it still remains controversial whether or not double T-tube decreases the risk of postoperative complications, long term follow-up was necessary to evaluate the outcomes of double T-tube method.

In current study, we evaluated clinical outcomes of 51 patients with FCBC who were treated either by double or single T-tube drainages. The main immediate postoperative complications were biliary leakage and fistulas, along with septic complications of the residual cavity leading to prolonged hospital stays in single T-tube group (*P* < 0.05)[16]. Most of these complications were ascribed to the pericyst lining the residual cavity. When a pericyst left in situ, especially thick and calcified ones, it would present an obstacle to liver regeneration and lead to abscess formation. Furthermore, the persistence of the pericyst hides possible biliary communication in the residual cavity, which has been considered as the main reason for biliary leakage [5, 6, 16]. However, it should be pointed out that even when biliary communications were identified, their closure within a stiff and calcified cystic wall would not be easy or effective. Furthermore, infection, biliary fistula, and slow reduction of the cyst cavity may contribute to more serious complications, such as obstruction of main hepatic ducts or the portal vein [1, 9, 11]. And, for this reason, we recommend a long-term follow-up, which was our another aim to report this study results after median 11 years observation. In these cases, reoperation is quite complexed, with high morbidities due to the technical difficulties related to distorted liver anatomy, deteriorated liver function, and poor general conditions of the patients. In our study, the overall stricture rate was 17.9% vs 0%, with late complications being more frequent in single vs. double T-tube drainage groups.

Although the longer follow-up period in double T-tube drainage group was necessary, no complications of stricture have been observed to date. Progressive shrinkage and fibrosis of the cystic cavity lead to development of traction in underlying bile duct which gets stenosed and resultant stricture formation. So, introducing the sustaining T-tube at the orifice of residual cavity, allowed the bile duct maintaining patency and being protected from postoperative strictures in double T-tube drainage group. We believed that after PPC and T-tube decompression, a further T-tube insertion into orifice of residual cavity bile duct was essential for the prevention of postoperative residual cavity infections and strictures especially when the location of the cyst was near to the hilum.

However, there were some failures in placing sustaining T-tube at FCBC site, or in other words, there could be alternative methods: (1) when corresponding upper level biliary tree was just a single or small-for-size liver segment, when there was liver atrophy in relevant liver parenchyma, when there was severe liver damage (would not be saved properly), a hepatectomy should be followed; (2) when it was difficult to place a sustaining T-tube or surgical suture, endo-drainage could be considered and would have to be managed by endoscopic approaches shortly after the surgery, but overall cost will increase and further evidences were needed; (3) when the FCBC site had an acute angle of sudden turn, it was hard to place sustaining T-tube. Based on our single center experience, we also propose possible indications for double T-tube method: (1) this should be individualized, not just only be dependent on FCBC size or location; (2) when the FCBC site is lobular ducts (1st or 2nd intrahepatic biliary tree) and near to hepatic hilum, or the duct was interrupted due to the rupture, a double T-tube drainage should be considered first; (3) when there is ulceration due to inflammation at the FCBC site, suturing may be difficult and double T-tube is recommended; (4) usually, > 5 mm diameter bile duct needs double T-tube drainage more than < 5 mm ones.

Shortcomings of this study was that it was a retrospective study based on relatively small sample size, and high-quality randomized case control studies would be very helpful to achieve better evidence-based results. In addition, removal timepoint should be optimized due to late leakage could happen at original FCBC site after withdrawal of decompression T-tube, in which occasion longer decompression was necessary. In addition, better study design with comparisons using endoscopic tools would be necessary in future studies [17–19].

## Conclusion

This research evaluated the advantages of additional sustaining T-tube of intrahepatic bile duct where the rupture occurred in the residual cavity. Although a longer follow-up period was necessary, this study revealed satisfactory follow up results of double T-tube drainage (decompression plus sustaining) compared to single T-tube drainage (decompression) after PPC for CE regarding long-term complications.

## Data Availability

The datasets used/analyzed during the current study are within the manuscript and more data could be available from the corresponding author on reasonable request.

## Abbreviations

CBD: Common bile duct
CE: Hepatic cystic echinococcosis
CT: Computed tomography
FCBC: Frank cysto-biliary complication
MR/MRCP: Magnetic resonance imaging and retrograde cholangiopancreatography
PPC: Partial peri-cystectomy

## References

[1] Wen H, Vuitton L, Tuxun T, Li J, Vuitton DA, Zhang W, McManus DP (2019). Echinococcosis: advances in the 21st century. Clin Microbiol Rev.

[2] Deplazes P, Rinaldi L, Alvarez Rojas CA, Torgerson PR, Harandi MF, Romig T, Antolova D, Schurer JM, Lahmar S, Cringoli G, Magambo J, Thompson RC, Jenkins EJ (2017). Global distribution of alveolar and cystic echinococcosis. Adv Parasitol.

[3] Brunetti E, Kern P, Vuitton DA (2010). Expert consensus for the diagnosis and treatment of cystic and alveolar echinococcosis in humans. Acta Trop.

[4] Akbulut S, Ozdemir F (2019). Intraperitoneal rupture of the hydatid cyst: four case reports and literature review. World J Hepatol.

[5] Tagliacozzo S, Miccini M, Amore Bonapasta S, Gregori M, Tocchi A (2011). Surgical treatment of hydatid disease of the liver: 25 years of experience. Am J Surg.

[6] Yildirgan MI, Basoglu M, Atamanalp SS, Aydinli B, Balik AA, Celebi F, Oren D (2003). Intrabiliary rupture in liver hydatid cysts: results of 20 years' experience. Acta Chir Belg.

[7] Shalayiadang P, Muzaffar I, Yusp Y, Turxun A, Nannan C, Wen H (2014). Comparison of post-operative short-term and long-term outcomes between occult and frank biliary rupture of hydatid disease. Hepatogastroenterology.

[8] Avcu S, Unal O, Arslan H (2009). Intrabiliary rupture of liver hydatid cyst: a case report and review of the literature. Cases J.

[9] Toumi O, Ammar H, Gupta R, Ben Jabra S, Hamida B, Noomen F, Zouari K, Golli M (2019). Management of liver hydatid cyst with cystobiliary communication and acute cholangitis: a 27-year experience. Eur J Trauma Emerg Surg.

[10] Pang Q, Jin H, Man Z, Wang Y, Yang S, Li Z, Lu Y, Liu H, Zhou L (2018). Radical versus conservative surgical treatment of liver hydatid cysts: a meta-analysis. Front Med.

[11] Akbulut S (2018). Parietal complication of the hydatid disease: comprehensive literature review. Medicine.

[12] Al-Saeedi M, Khajeh E, Hoffmann K, Ghamarnejad O, Stojkovic M, Weber TF (2019). Standardized endocystectomy technique for surgical treatment of uncomplicated hepatic cystic echinococcosis. PLoS Neglect Trop Dis.

[13] Erzurumlu K, Dervisoglu A, Polat C, Senyurek G, Yetim I, Hokelek M (2005). Intrabiliary rupture: an algorithm in the treatment of controversial complication of hepatic hydatidosis. World J Gastroenterol.

[14] Kayaalp C, Bostanci B, Yol S, Akoglu M (2003). Distribution of hydatid cysts into the liver with reference to cystobiliary communications and cavity-related complications. Am J Surg.

[15] Yang H, Tang J, Peng X, Zhang S, Sun H, Lv H, Li J, Chen X (2012). Treatment of complicated hepatic cystic hydatidosis with intrabiliary rupture by pericystectomy in combination with Roux-en-Y hepaticojejunostomy. J Huazhong Univ Sci Technol Med Sci.

[16] Boyce DSK, Ellis JS, Hightower SL, Lew JL, Price MW, Lin-Hurtubise KM, Hostler JM (2019). Recurrent inactive hydatid cyst of the liver causing restrictive pulmonary physiology. Hawai'i J Health Soc Welfare.

[17] Kemal D, Sami A (2014). Role of endoscopic retrograde cholangiopancreatography in the management of hepatic hydatid disease. World J Gastroenterol.

[18] Yasar N, Metin K, Sami A (2015). Role of chemotherapeutic agents in the management of cystic echinococcosis. Int Surg.

[19] Cemalettin K, Sami A, Tevfik TS, Adem T, Sezai Y (2020). Intraperitoneal rupture of the hydatid cyst disease: single-center experience and literature review. Ulus Travma Acil Cerrahi Derg.

